# The Thyrohyoid Syndrome: Promoting Awareness with a Case Report and Systematic Review of the Literature

**DOI:** 10.3390/diagnostics14121227

**Published:** 2024-06-12

**Authors:** Raphael Jeker, Linda März, Lukas Horvath

**Affiliations:** Department of Otorhinolaryngology, Head and Neck Surgery, Kantonsspital Aarau, 5001 Aarau, Switzerland

**Keywords:** cervical pain, neck pain, odynophagia, steroid injection, thyrohyoid ligament

## Abstract

Objective: Neck pain is commonly referred to an ENT specialist and can be caused by the little-known inflammatory condition of the lateral thyrohyoid ligament. The pathophysiology of this condition is believed to be inflammation subsequent to over-exertion or cervical trauma. Typically, patients present with chronic unilateral neck pain. Elicitation of localized tenderness over the axis of the lateral thyrohyoid ligament on palpation is a key finding for its diagnosis. We present an unusual case with an acute course and subcutaneous inflammation and discuss its management in an effort to raise awareness for this often-misdiagnosed syndrome. Methods: A systematic literature research on PubMed was performed selecting patients with a definitive diagnosis of thyrohyoid syndrome or lateral thyrohyoid ligament syndrome. Results: We collected 54 cases from three studies. This condition is an important differential diagnosis for acute or chronic antero-lateral or unilateral neck pain. Conclusion: No specific radiological findings are defined and a CT scan is therefore not necessary for its diagnosis, but ultrasound is a useful tool to primarily assess any neck lesion. Once the diagnosis is made, a local infiltration of steroids is the most sustainable treatment option and relapse prevention.

## 1. Introduction

Acute or chronic neck pain is a common reason for referral to an ENT specialist. The thyrohyoid syndrome presents as such and is a little-known inflammatory condition of the lateral thyrohyoid ligament and nearby tissues. It is distinctive from the well-known hyoid- or eagle syndrome [1]. We chose to present this little-known condition as a case report to promote awareness among health care workers with the goal of increasing correct diagnosis and treatment.

In the scarcely available literature, a 2:1 female predominance as well as a mean age of 55 years is reported. Currently there are no published systematic reviews on this topic.

In regards to its pathogenesis, irritative inflammation of the ligamentous, cartilaginous or bursa-related anatomical structures of the thyrohyoid region is believed to be the cause of the symptoms and can be triggered by overuse or factors such as a cough, voice abuse or strenuous neck and upper limb movements [1]. The first mention of this syndrome in the literature cannot be defined because imprecise and unstandardized nomenclature has been used in the past.

The terms thyrohyoid syndrome or lateral thyrohyoid ligament syndrome describe the condition most accurately. Other terms, such as hyoidynia or hyoid bursitis have also been used to describe local inflammatory conditions of the hyoid region. The closely related term “hyoid syndrome” has been used in 1975 by Kopstein to describe a multitude of conditions relating to the hyoid bone [2,3].

## 2. Detailed Case Description

**Figure 1:**
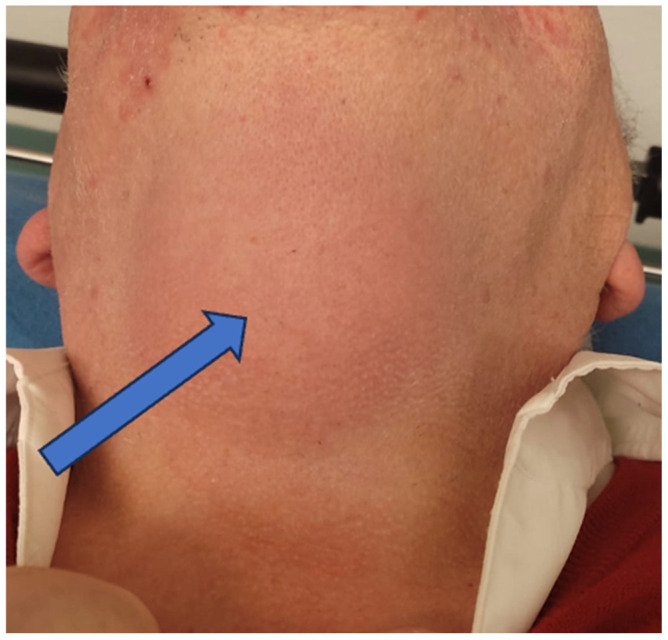
Clinical presentation with erythematous skin as a sign of local cervical inflammation. Arrow pointing to the location of maximal palpatory tenderness.

**Figure 2:**
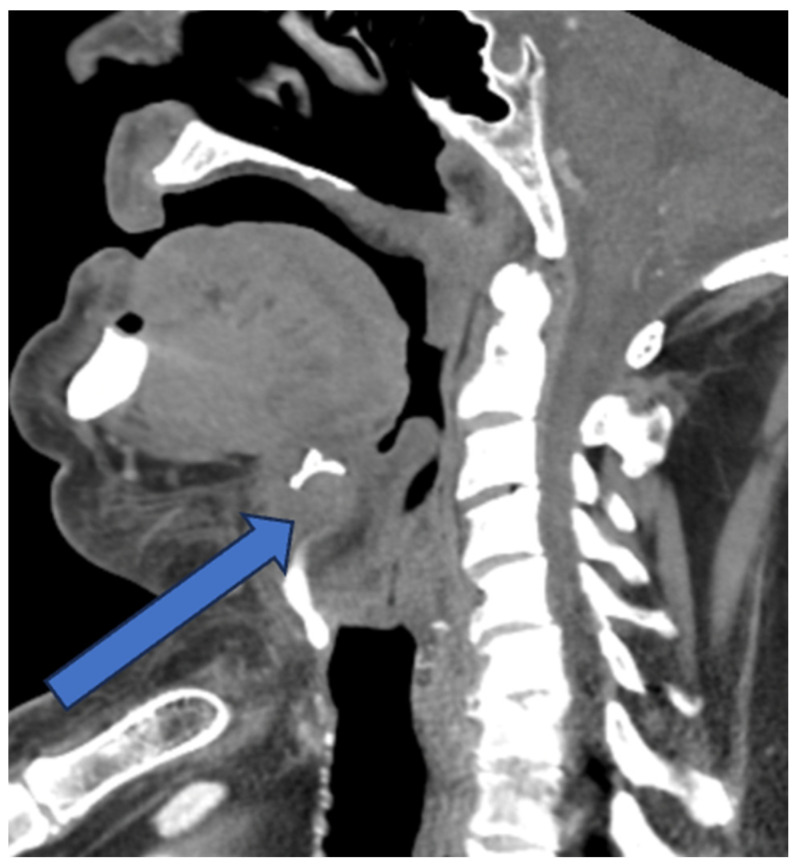
Sagittal view on a CT scan showing unspecific inflammatory lesion of the soft tissue. Arrow pointing to the thyrohyoid membrane and the hyoid bone with peripheral tissue inflammation.

A 74-year-old man presented to the Emergency Department with a two-day history of swelling and erythema of the anterior cervical area without fever, spontaneous pain or cervical tightness. However, odynophagia was present with minor right dominant pain on palpation of the thyrohyoid complex. The personal history revealed diverticulosis, an active smoker of 50 pack years and no current medication. On physical examination the patient presented with an erythematous and warm swelling extending over an area of 7 × 7 cm from submental to the thyroidal cartilage with minimal right dominance (Figure 1). The remaining ENT examination including flexible nasal laryngoscopy was unremarkable. The blood work showed elevated inflammatory parameters (Lc 6 G/L, CRP 80 mg/L). On ultrasound of the neck, hyperechogenic swelling of the soft tissues in the anterior cervical region (Levels Ia and VI) with ill-defined borders and singular hypoechogenic foci were visible. A computed tomography (CT) scan of the neck showed a median anterior cervical right-dominant thyro-hyoidal lesion with discrete radiocontrast enhancement along the thyrohyoid membrane and the hyoid bone with peripheral tissue inflammation but no abscess (Figure 2).

Initially, empiric therapy with broad-band antibiotics and non-steroidal anti-inflammatory drugs (NSAIDs) was administered. After ruling out abscess formation or phlegmon, systemic corticosteroids were given in addition. Subsequently, the cervical erythema and swelling as well as the elevated inflammation markers declined.

**Figure 3:**
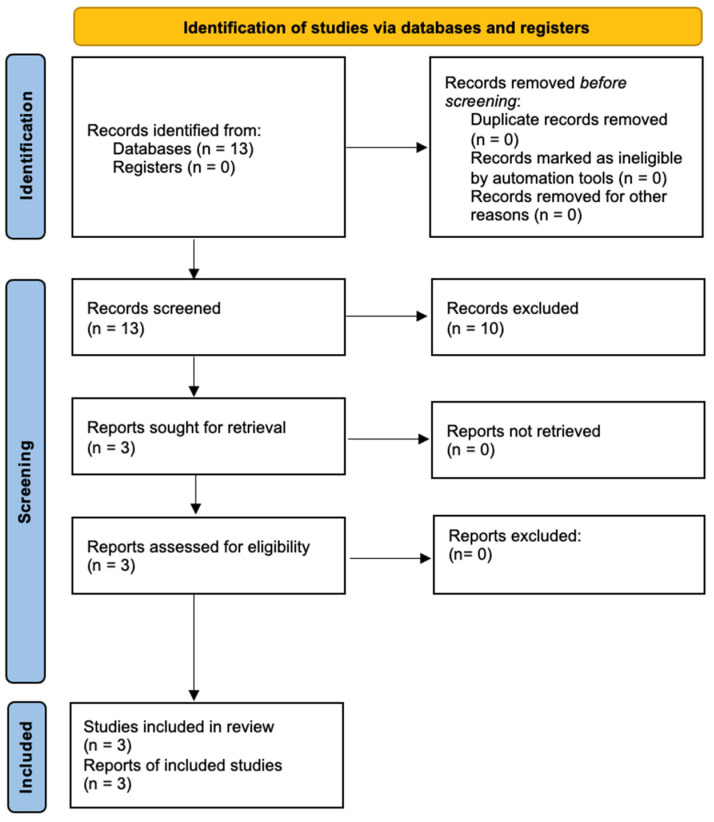
Flow diagram of the literature search, based on the PRISMA statement 2020.

A review of the literature was conducted using PubMed based on the PRISMA statement until 1. October 2023. A key word search using the words “thyrohyoid syndrome”, “thyro-hyoid syndrome”, “thyroid ligament syndrome” and “lateral thyrohyoid ligament syndrome” was performed identifying only 13 studies. Of those results, only three records referred to the treatment and discussion of the thyrohyoid syndrome. The excluded publications regarded other diseases causing neck pain and their respective treatment, e.g., the hyoid syndrome, the clicking larynx syndrome or the eagle syndrome. No further search restrictions were applied. An effort was made to identify relevant articles through hand searching from reference reviews. An additional literature review was carried out on similar conditions like the “hyoid syndrome” and “eagle syndrome” using a comparable process. The data of patients included in the review were analyzed by descriptive statistics (Figure 3).

We found reports between 2002 and 2023 on a total of 54 patients in three different studies. In total, there were 36 females and 18 males, with a mean age of 55 years. There was an overall 2:1 female predominance. The majority presented as chronic neck pain (>3 month). For diagnostic reasons, 30 patients (56%) initially received a CT scan to rule out other threatening pathologies, although no specific radiologic findings exist for its diagnosis. Treatment included either systemic or local steroids. Thirty-nine patients (72%) received local steroid (triamcinolone) injections and fifteen (28%) subjects were treated with systemic steroids. Both groups experienced significant pain relief, but local steroid injections were found to be superior to prevent relapse in the long term (Table 1).

## 3. Discussion

**Figure 4:**
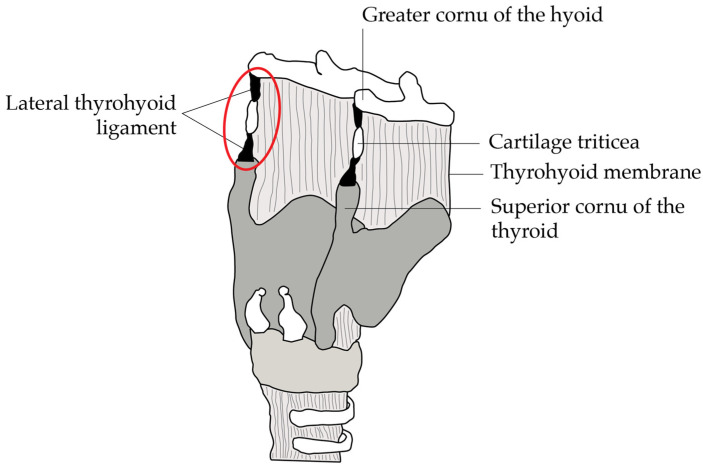
Posterolateral view of the anatomical structures. Target injection site for treatment is shown by the circle. Figure adapted from Sinha et al. [1].

The thyrohyoid syndrome is an important differential diagnosis for chronic antero-lateral or unilateral neck pain and its associated symptoms are believed to originate from an inflammation of unknown cause of the thyrohyoid ligament, its insertion points, the incorporated cartilago triticea and the nearby Boyer’s bursa with subsequent calcification and tendinitis [1,4] (Figure 4).

Our patient presented in a rare acute manner with slight induration, tenderness and erythematous skin with ill-defined borders at the antero-lateral neck region. This prompted the administration of an empiric antibiotic treatment and performance of an ultrasound and CT scan to rule out a deep neck infection. However, there are no specific diagnostic radiologic findings in the diagnosis of the thyrohyoid syndrome [1]. We concluded that our patient additionally exhibited subcutaneous inflammation, possibly due to secondary local infection, but clearly showed the pathognomonic point of acute tenderness over the greater horn of the hyoid bone. A possible origin of the inflammation is believed to be subsequent to placing stress on the connective tissues by voice abuse, swallowing, chronic cough, neck and upper limb overuse or cervical trauma [5]. A history of antero-lateral or lateral neck pain, odynophagia and foreign body sensation confirms the diagnosis along with finger or thumb pressure to find the pathognomonic point of acute tenderness over the greater horn of the hyoid bone and sometimes also the upper border of the thyroid cartilage.

Numerous conditions are described in association with neck pain and need to be considered as differential diagnosis, ranging from degenerative disease, neck trauma, congenital malformations to infections and malignancy. In this regard, all neck structures need to be taken into consideration including muscles and compartments consisting of vertebral, visceral and vascular compartments. Within the latter, the neck houses soft tissue structures, salivary glands, the thyroid and parathyroid, nerves, arteries and veins, lymphatic system, vertebrae, discs and the cervical spinal cord, as well as the upper respiratory and digestive tract. In the presented case, due to its acute presentation, clinical pictures such as an infected thyroglossal or branchial cyst, erysipelas, deep neck infection or Lemierre syndrome, lymphadenopathy, thyroiditis or myositis were also deliberated. Most of the mentioned considerations were ruled out following clinical examination and neck ultrasound. Generally, the ultrasound should be the first-line diagnostic imaging tool for laryngeal and neck lesions due to its availability [6,7]. Magnetic resonance imaging is usually not available in an emergency setting. In this presented case, the CT scan served as an additional immediate diagnostic tool pointing to the final diagnosis. However, it is likely that if our patient had presented without acute pain, neck erythema and induration, a CT scan would not have been initiated, thus leading the treating doctors to an approximate diagnosis by eliciting localized tenderness over the lateral thyrohyoid ligament axis. In such a manner, a refined differential diagnosis represents hyoid bone insertion tendinitis, retropharyngeal calcific tendinitis, scalenus anticus syndrome, Eagle’s syndrome, superior laryngeal neuralgia and carotidynia [8,9].

In histological analyses of specimens from patients with thyrohyoid syndrome, degenerative damage to the hyoid bone and adjacent structures have been found [5]. Baseline treatment with NSAIDs has found unsatisfactory results in symptom control in patients with thyrohyoid syndrome, especially in patients reporting symptoms >6 weeks (66% symptom improvement) [5]. On the other hand, a more invasive attempt with surgical excision of the hyoid bone has been tried in the past, similarly not yielding a satisfactory outcome despite its invasiveness, since complete resolution was only achieved in 83.3% of cases [5]. Recently, systemic as well as local injection of corticosteroid has become the therapy of choice [5]. In the available literature with systemic corticosteroid treatment, 86.7% reported a partial or full resolution compared to 90% reporting symptom resolution after local injection treatment. In the 1- to 5-year follow-up of patients treated with local injection, no recurrence was reported. Kunjur et al. [5] proposes an injection of 0.5 mL Kenalog (40 mg per mL) at the lateralmost point of the hyoid and upper border of the thyroid cartilage (Figure 4).

We present the first systematic review of the little-known thyrohyoid syndrome, creating awareness of its chronic and acute presentation. The limited available data in the literature as well as the low number of patients in the referenced papers on this condition presented a significant challenge and pose a limitation to this study. Another limitation is the dissimilar and divided definitions of localized inflammatory conditions in the antero-lateral neck region.

## 4. Conclusions

The thyrohyoid syndrome is a condition that must be considered by otolaryngologists during the evaluation of patients with antero-lateral neck pain in the thyrohyoid region. It is important to note that most cases present as chronic unilateral neck pain and that the pain does usually not resolve over time [1,5]. The pain is worsened by neck movement, swallowing, chewing and sometimes speaking [10]. However, in rare cases, the inflammation of the thyrohyoid region can also present in an acute manner, mimicking more serious neck conditions. In such cases, differential diagnoses as discussed above must be taken into consideration.

The investigation of the thyrohyoid syndrome is made by a thorough medial history and performing clinical examination of the head and neck, including a flexible laryngoscopy. Elicitation of localized tenderness over the lateral thyrohyoid ligament axis on palpation is a key clinical finding for its diagnosis. Usually, a CT scan is not necessary but sonographic imaging is a useful tool to rule out other pathologies, while not exposing the patient to any radiation [6]. While the administration of a short course of oral steroids and NSAID is feasible, the most promising and sustainable treatment option is the local infiltration of steroids and anesthetics.

## Figures and Tables

**Table 1:** Overview of the relevant literature with clinical presentation, applied diagnostic measures, treatment modalities, sex and age of the patient collection. Dx: diagnostic tool; CT: computer tomography; Sono: sonography/ultrasound.

Study	Chronic (>3 Month)	Acute	Dx Clinical	Dx CT	Dx Sono	Local Steroids	Systemic Steroids	Male	Female	Mean Age
Kunjur et. al. [4]	13	0	13	0	0	13	0	5	8	54
Sinah et. al. [1]	15	0	15	4	0	15	0	6	9	57
Choi et. al. [5]	26	0	26	26	0	11	15	7	19	42.9/39.5

## Data Availability

The references of all included cases as well as datasets generated and analyzed during the present study are available from the corresponding author upon reasonable request.
